# Improving the diagnosis of central nervous system infections: experience with the off-label use of FA-BCID2

**DOI:** 10.1128/spectrum.03189-25

**Published:** 2026-01-21

**Authors:** Giulia Menchinelli, Margherita Cacaci, Damiano Squitieri, Giulia De Angelis, Maurizio Sanguinetti, Brunella Posteraro

**Affiliations:** 1 Dipartimento di Scienze di Laboratorio ed Ematologiche, Fondazione Policlinico Universitario A. Gemelli IRCCS, Rome, Italy; 2 Dipartimento di Scienze Biotecnologiche di Base, Cliniche Intensivologiche e Perioperatorie, Università Cattolica del Sacro Cuore, Rome, Italy; 3 Unità Operativa “Medicina di Precisione in Microbiologia Clinica”, Direzione Scientifica, Fondazione Policlinico Universitario A. Gemelli IRCCS, Rome, Italy; University of Maryland School of Medicine, Baltimore, Maryland, USA

**Keywords:** meningitis, molecular syndromic panel, multiplex PCR assays

## LETTER

Multiplex PCR panels for central nervous system (CNS) infections are widely used but have well-recognized limitations: the FilmArray Meningitis/Encephalitis (FA-ME) panel (bioMérieux, Marcy l’Étoile, France) is strong for “ruling in” yet limited for “ruling out,” and negative results do not exclude infection (1–3).

We report our experience with the off-label use of the FilmArray Blood Culture Identification 2 (FA-BCID2) panel (bioMérieux) on cerebrospinal fluid (CSF), focusing on bacterial pathogens relevant to hospital-acquired (HA) CNS infections and late-onset neonatal meningitis (e.g., *Klebsiella pneumoniae*, *Enterobacter cloacae* complex, *Acinetobacter baumannii* complex, and coagulase-negative staphylococci) in contrast to FA-ME, which primarily targets community-acquired (CA) bacteria (4). Previous small studies on FA-BCID/BCID2 in CSF have reported promising performance (5–7).

This study was performed at a large tertiary-care university hospital with neurosurgical services and analyzed two periods, that is, before and after implementation of FA-BCID2. We included all culture-positive bacterial meningitis cases for which at least one FilmArray assay (FA-ME or FA-BCID2) was performed: 79 cases in the pre-implementation period and 34 in the post-implementation period. These cases represented 11.2 (79/703) and 9.5% (34/358) of all CSF samples for which a FilmArray assay was performed for suspected meningitis in the respective periods. In the post-implementation period, infectious disease specialists requested molecular testing for suspected meningitis by phone and indicated whether the case was adult CA or HA or neonatal early- versus late-onset using standard clinical and epidemiological criteria consistent with the corresponding published definitions (8, 9). Based on this classification, CSF samples were tested with FA-ME for CA/viral differential diagnoses and with FA-BCID2 for suspected HA infections. FA-BCID2 was used off-label directly on CSF (no enrichment) after internal verification using contrived/spiked CSF and known-positive samples.

**Fig 1:**
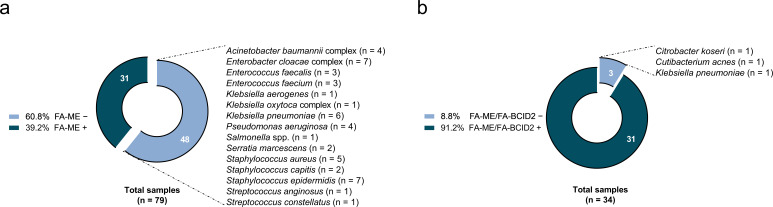
(**a**) Pathogen distribution in CSF culture-positive cases detected by the FilmArray Meningitis/Encephalitis (FA-ME) panel (May 2018–April 2023). (**b**) Pathogen distribution after implementation of the combined FA-ME + FilmArray Blood Culture Identification 2 (FA-BCID2) panel algorithm (May 2023–June 2025). “+” and “–” indicate positive and negative molecular assay results, respectively.

In the pre-implementation period (May 2018–April 2023), FA-ME was the only molecular assay used; there were 79 culture-confirmed bacterial meningitis cases (HA 41, CA 28, neonatal 10), and FA-ME detected the pathogen in 31/79 (39.2%). The 48 FA-ME-negative results represented missed opportunities for early targeted antimicrobial therapy. In the post-implementation period (May 2023–June 2025), a combined algorithm with FA-ME and FA-BCID2 was adopted; culture-positive cases were 34 (HA 16, CA 13, late-onset neonatal 5), with an overall molecular detection of 31/34 (91.2%). Excluding the 13 CA cases (all correctly detected by FA-ME), 14/16 (87.5%) HA and 4/5 (80.0%) neonatal late-onset cases were correctly detected by FA-BCID2. Notably, two neurosurgical patients—one with *K. pneumoniae*, the other with *Cutibacterium acnes* meningitis—tested negative with FA-BCID2 and were confirmed only by 16S rRNA PCR/sequencing, while *Citrobacter koseri* meningitis in a neonate was missed as an off-panel pathogen. The corresponding pathogen distributions and detection rates by period and category are shown in Fig. 1.

While the molecular detection rate improved markedly, the clinical impact of this strategy (e.g., faster initiation of targeted therapy or improved patient outcomes) was not assessed in this study.

In conclusion, our data suggest that the combined use of FA-ME and FA-BCID2 assays may improve the molecular diagnosis of HA bacterial meningitis and late-onset neonatal meningitis. However, false negatives and off-panel organisms underscore that these panels should complement, rather than replace, conventional culture and highlight the need for molecular assays specifically tailored to nosocomial CNS infections. Revisiting diagnostics in this field means not only introducing new assays but also rethinking the scope and adaptation of existing ones.
